# USE OF WARFARIN IN PEDIATRICS: CLINICAL AND PHARMACOLOGICAL CHARACTERISTICS

**DOI:** 10.1590/1984-0462/;2017;35;4;00008

**Published:** 2017-09-21

**Authors:** Bruna Bergmann Santos, Isabela Heineck, Giovanna Webster Negretto

**Affiliations:** a Universidade Federal do Rio Grande do Sul, Porto Alegre, RS, Brasil.; b Hospital de Clínicas de Porto Alegre, Porto Alegre, RS, Brasil.

**Keywords:** Warfarin, Children, Thrombosis, Anticoagulants, Varfarina, Crianças, Trombose, Anticoagulante

## Abstract

**Objective:**

To describe how children respond to oral anticoagulation with warfarin, verifying the influence of age, clinical condition, route of administration of warfarin and use of total parenteral nutrition (TPN), as well as to describe risk factors for the occurrence of thrombotic events (TE) in childhood.

**Methods:**

A retrospective descriptive study including all patients ≤18 years old for whom warfarin was prescribed in a university hospital. Patients were divided according to clinical condition, age, route of medication administration and use of TPN. Data was collected from the patients’ medical records and the analysis considered the risk factors for TE already described in the literature, the time and the dose required in order to reach the first International Normalized Ratio (INR) in the target and the adverse events in this period. After reaching the INR, the maintenance of anticoagulation was verified by the prescribed dose and INR tests.

**Results:**

Twenty-nine patients were included in the study. The major risk factor for TE was the use of a central venous catheter in 89.6% of the patients. Patients with short bowel syndrome and total parenteral nutrition required significantly higher doses (*p*≤0.05) to achieve and maintain the INR in the target. Patients ≤1 year old needed longer periods and required an increased dose of anticoagulation and maintenance than older patients. The mean number of INR examinations below the target was 48.2% in the groups studied.

**Conclusions:**

The observed complexity of anticoagulant therapy reinforces the need to develop protocols that guide clinical practice.

## RESUMO

**Objetivo:**

Descrever como as crianças respondem à anticoagulação oral com varfarina, verificando a influência da idade, da condição clínica, da via de administração da varfarina e do uso de Nutrição Parenteral Total (NPT), e apresentar a presença de fatores de risco para eventos tromboembólicos (TE).

**Métodos:**

Estudo transversal retrospectivo com pacientes ≤18 anos que iniciaram o uso da varfarina em um hospital universitário. Os pacientes foram divididos conforme condição clínica, idade, forma de administração do medicamento e uso de NPT. Foram utilizados os dados dos prontuários dos pacientes, considerando os fatores de risco para TE já descritos na literatura, o tempo e a dose necessária para atingir a primeira Razão Normalizada Internacional (INR) no alvo e eventos adversos nesse período. No período posterior ao alcance de INR, foi verificada a manutenção da anticoagulação, por meio da dose prescrita e dos exames de INR.

**Resultados:**

Vinte e nove pacientes foram incluídos no estudo. O principal fator de risco para TE foi o uso de cateter venoso central, em 89,6% dos pacientes. Os pacientes com síndrome do intestino curto e em uso de NPT necessitaram de doses significativamente maiores (*p*≤0,05) para atingir e manter a INR no alvo. Os pacientes com ≤1 ano levaram mais tempo e necessitaram de uma dose maior para anticoagular e para manter o INR no alvo que os pacientes mais velhos. A mediana de exames de INR abaixo do alvo foi de 48,2% nos grupos estudados.

**Conclusões:**

A complexidade da terapia anticoagulante reforça a necessidade da elaboração de protocolos que orientem a prática clínica.

## INTRODUCTION

Warfarin is the most frequent anticoagulant used in pediatrics. Its use has become more and more common due to the increasing number of thrombotic events (TE) in this population owed to the increasing survival rates of children with severe conditions.^1^ These events are under-notified in children, and occur less frequently than among adults because of protective physiological mechanisms, such as the lower capacity to generate thrombin, the higher capacity of alpha-2 macroglobulin to inhibit thrombin, and the higher antithrombotic potential of the blood vessel wall.^1^ ^,^ ^2^ More than 90% of these events are associated with some risk factor^1^ and are more common among children with severe conditions, such as cancer and the short bowel syndrome (SBS). These diseases often lead to the prolonged use of a central venous catheter (CVC) to administer total parenteral nutrition (TPN), chemotherapy or antimicrobial therapy.^3^ ^,^ ^4^ ^,^ ^5^ Besides, it is known that oncology patients present other predisposing factors to thrombosis, such as the tumor itself, immobilization and the need for surgeries.^6^

The use of warfarin in children points out to many peculiarities. While children are growing, there are many changes in the hemostatic system, so the dose and the handling depends on age.^7^ In this population, it is more frequent to see clinical intercurrences, changes in diet, use of drugs that interact with warfarin reported in the literature and the need for a pharmaceutical derivation using pills, due to the unavailability of the pharmaceutical form and doses that are adequate to the age group. All these factors make it difficult to handle anticoagulation, and lead to the need for more monitoring.^1^ ^,^ ^8^ ^,^ ^9^ The most common test used to monitor the warfarin therapy is the prothrombin time (PT) test, reported by the International Normalized Ratio - INR).

Warfarin is considered as a potentially dangerous medication by the Institute for Safe Practices in the Use of Drugs (ISMP). Some of its characteristics are: the wide range of dose-response, the narrow therapeutic window and the high risk of adverse events, like bleeding.^10^ Because of the lack of data and the few clinical studies and recommendations about the use of warfarin in children, there is extrapolation of data from clinical trials conducted with adults, even if there are hemostatic differences between the adult and the pediatric population.^8^ ^,^ ^11^

In this context, this study aims at describing the characteristics of the use of warfarin in pediatrics and at obtaining information about the way factors such as age, clinical condition, route of warfarin administration and use of TPN can influence the response to treatment, as well as describing the presence of risk factors for thrombosis.

## METHOD

This is a cross-sectional study that was conducted in a university hospital of Porto Alegre, approved by the Ethics Committee, report n. 1.438.563. The patients were selected from the database of the Pharmaceutical Follow-up in the Clinical Pharmacy Section from the Pharmacy Service referring to the period of January, 2014, to September, 2015. The study included all pediatric patients (0 to 18 years) who were hospitalized in the pediatric units of the hospital and who started on warfarin in this period. Those using the medication for insufficient time to obtain the data about the anticoagulation process (<1 week) and therapeutic error (non-coagulation) were excluded. Medical prescriptions, test results, records of hospital admissions and outpatient appointments corresponding to the study period were accessed retrospectively, through an electronic medical record.

The data were collected using a data collection form. Risk factors for TE already studied in the literature were analyzed, such as: presence of CVC, use of TPN, sepsis, surgeries, thrombophilia, cancer, among others.^12^ ^,^ ^13^ The first part of collection corresponded to the first dose of warfarin until the moment when the patient reached the first INR on the target, being classified as anticoagulated. INR target considered in the study was 2.5, and the therapeutic range, between 2 and 3, as indicated by the literature and according to the objective of the medical team for the studied patients.^8^ In this period, the following was assessed: time (in days) required for anticoagulation, dose of anticoagulation, concomitant use of other anticoagulants, occurrence of altered INRs (>4), occurrence of bleeding and fixed prescribed medicines. Based on the data about the fixed medications used in medical prescriptions, it was possible to identify the potentially severe drug interactions with good documentation (good evidence of occurrence, coming from well-controlled studies or with multiple case reports), using the software Drug-Reax (Thomson Micromedex, Greenwood Village, Colorado, United States). The second stage corresponded to the post-anticoagulation period until the end of the study period, when all INR and dose (mg/kg) values were verified to determine the permanence of INR in the therapeutic range (Target Therapeutic Range - TTR), and the dose of treatment maintenance.^14^

For the organization of the data, a base was developed using the Excel software, version 2010. The data were analyzed in the Statistical Package for the Social Sciences (SPSS), version 18.0 for Windows (SPSS Inc., Chicago, IL, United States). Descriptive statistical analysis was used with absolute and median frequencies, and an interquartile range. Considering the number of patients and the distribution of data, the non-parametric Kruskal-Wallis and the Mann-Whitney U test were used for group comparison. Values of p≤0.05 were considered significant.

## RESULTS

Thirty-two patients (54.8% male participants) aged between 1 month and 18 years began using warfarin in the studied period. Three patients were excluded from the study: two because of insufficient time of use to obtain the data about the anticoagulation process, and one for therapeutic error. Of the 29 patients left, two patients received two warfarin routes due to a new TE, thus resulting in a total of 31warfarin routes.

All warfarin routes (31) were prescribed for secondary prophylaxis to venous thromboembolism (VTE), whose occurrence was 64.5% in the upper venous network, 6.5% in the upper and lower venous network, 6,5% in the portal vein, 12.9% in the NCS and 9.7% in other places.

**Graph 1:**
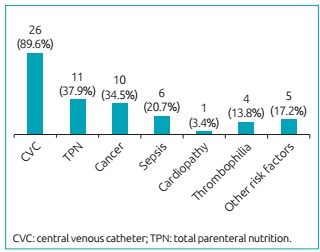
Presence of each risk factor for thromboembolic events in the patients of the study.

Graph 1 shows the risk factors observed in the 29 patients when they had a thrombotic event. The risk factor that was most present was the use of CVC, found in 89.6% of the patients, followed by TPN (37.9%), cancer (34.5%), sepsis (20.7%), cardiopathy (3.4%) and other risk factors, such as surgery and infections (17.2%). The presence of thrombophilia was investigated in 12 patients, of whom four (13.8%) presented at least one thrombophilia: three had protein S clotting disorder (free antigen), one presented mutation in Factor V Leiden, and another one had immunoglobulin G anticardiolipin (IgG) and immunoglobulin M (IgM). Three recurrences of thrombosis were observed, being one in the validity of the anticoagulant, and two without using warfarin.

Table 1 contains the data of the 31 medication routes grouped, considering the factors that could interfere in the anticoagulation process: clinical condition (cancer, SBS and other comorbidities), age, route of drug administration, and use of TPN. The number of warfarin routes are presented, as well as age and time of treatment for each group, expressed in median and interquartile range. There were no significant differences between the groups regarding these characteristics.

**Table 1:**
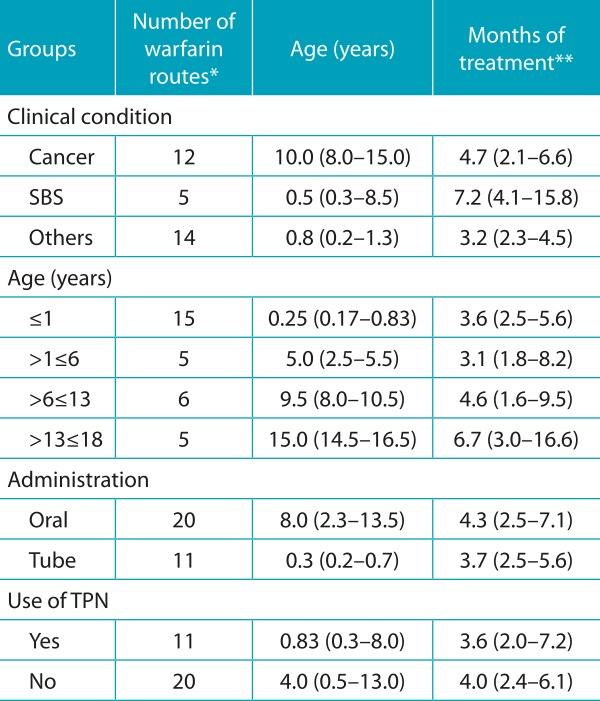
General characteristics of the 29 patients and 31 routes studied, expressed in median and interquartile range.

**Four patients continued using after the end of the study. SBS: Small bowel syndrome; TPN: total parenteral nutrition.

Table 2 has the data about the anticoagulation process. Younger patients (≤1 year) required a larger dose for anticoagulation (median: 0.30 mg/kg; interquartile range: 0.10-0.40 mg/kg) in comparison to the other groups. Besides, in infants aged 1 year or less, the dose initially prescribed was significantly higher than for the others (*p*=0.017). Patients with SBS required initial doses of warfarin (median: 0.40 mg/kg; interquartile range: 0.30-0.40 mg/kg) and doses for anticoagulation (0.40 mg/kg; 0.40-0.43 mg/kg), significantly higher than for patients who presented with other conditions (*p*=0.019 and *p*=0.025, respectively). Likewise, the use of TPN was associated with the initial dose (median 0.40 mg/kg; interquartile range: 0.20-0.40 mg/kg) and with the anticoagulant dose (0.40 mg/kg; 0.33-0.45), significantly higher than other forms of nutrition (*p*=0.003 and *p*=0.002, respectively). Patients using TPN and, oral administration, had the longest mean time for anticoagulation; however, the differences observed regarding time were not significant.

**Table 2:**
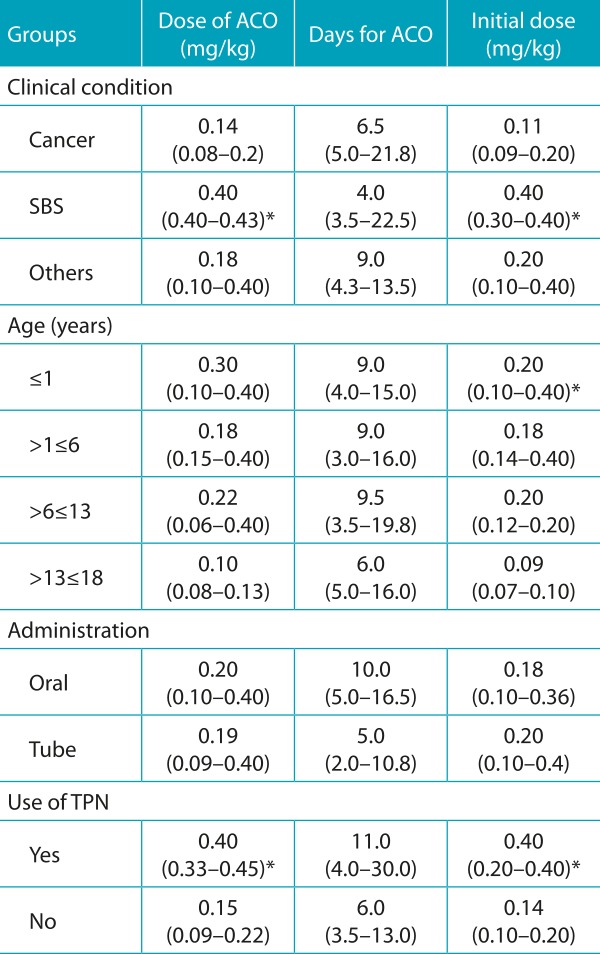
Data about the anticoagulation process for each analyzed group, expressed in median and interquartile range. Total assessed: 31 routes in 29 patients.

SBS: small bowel syndrome; TPN: total parenteral nutrition; ACO: anticoagulation; * Kruskal-Wallis and Mann-Whitney U Tests (p≤0,05).

During the anticoagulation period, in seven (22.6%) routes, patients presented with INR>4, and one of them had intestinal bleeding. In this period, in 29 warfarin routes, patients used unfractioned heparin (UFH) simultaneously, and/or low-molecular-weight-heparin (LMWH). Out of these, in 18 routes heparin was used until anticoagulation.

Table 3 refers to the data after anticoagulation and shows how long patients stayed anticoagulated. Two warfarin routes were excluded from this analysis due to the suspention of the drug right after anticoagulation; one because the treatment was over after three months, and another one to conduct a surgical procedure, not restarting its use until the end of the study. Therefore, 29 warfarin routes were analyzed.

**Table 3:**
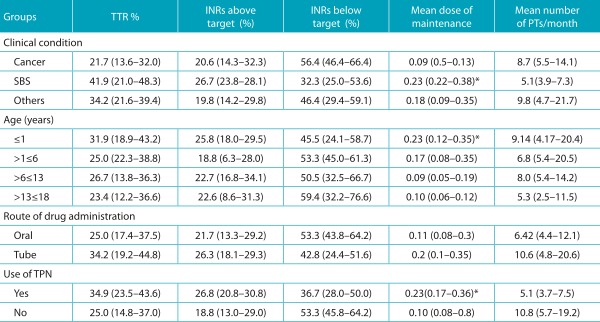
Data of the monitoring of warfarin therapy (31 routes in 29 patients), reported by the TTR^14^, mean dose of maintenance and number of test requests per month, expressed in median and interquartile range.

*Kruskal-Wallis and Mann-Whitney U test (p≤0.05). Two patients did not undergo anticoagulation monitoring because the drug was suspended right after reaching the first INR on the target, so both were excluded from this analysis (n=29). SBS: small bowel syndrome; TPN: total parenteral nutrition; ACO: anticoagulation; TTR: target therapeutic range; INR: international normalized ratio; PT: prothrombin time.

Patients with SBS had higher TTR (median: 41.9%; interquartile range: 21.0-48.3%) when compared to patients with cancer (21.7%; 13.6-32.0%) and with other conditions (34.2%; 21.6-39.4%). Younger patients (median: 31.9%; interquartile range: 18.9-43.2%) had more INRs in the therapeutic range than older groups (23.4%; 12.2-36,].6%). The same was observed for patients who administered the drug via nasoenteric tube (median: 34.2%; interquartile range: 19.2-44.8%), compared to the oral administration (25%; 17.4-37.5%), and also the ones using TPN (34.9%; 23.5-43.6%) and the ones not using it (25.0%; 14.8-37.0%). The median of INR tests below the target was 48.2% in the studied groups. In all groups, patients presented more INRs below the target than above it, or within the therapeutic range.

Patients with SBS required a larger dose of warfarin to maintain the INR in the target (median: 0.23 mg/kg; interquartile range: 0.22-0.38 mg/kg) than patients with cancer (0.09 mg/kg; 0.05-0.13 mg/kg) and with other conditions (0.18 mg/kg; 0.09-0.35 mg/kg), with significant difference (*p*=0.007). Younger patients (≤1 year) also required a significantly higher dose (*p*=0.044) of warfarin (median: 0.23 mg/kg; interquartile range: 0.12-0.35 mg/kg) in relation to older ones (0.10 mg/kg; 0.06-0.12 mg/kg). The administration of the drug via nasoenteric tube and the use of TPN were associated with a higher dose of anticoagulant maintenance [(0.20 mg/kg; 0.10-0.35 mg/kg) and (0.23 mg/kg; 0.17-0.36 mg/kg), respectively]. The difference in doses observed between the groups that used TPN or not was also statistically significant in this post-anticoagulation period (*p*=0.024).

The scientific literature classifies drug interactions according to the severity and type of documentation.^15^ In the anticoagulation period, 69 potential drug interactions involving warfarin were found, and these interactions were considered severe, with good documentation. Among the drugs mostly prescribed, which could present drug interactions, it is possible to mention the following: sulfamethoxazole + trimethoprim (18.8%), fluconazole (17.4%) e metronidazole (14.5%), according to Table 4.

**Table 4:**
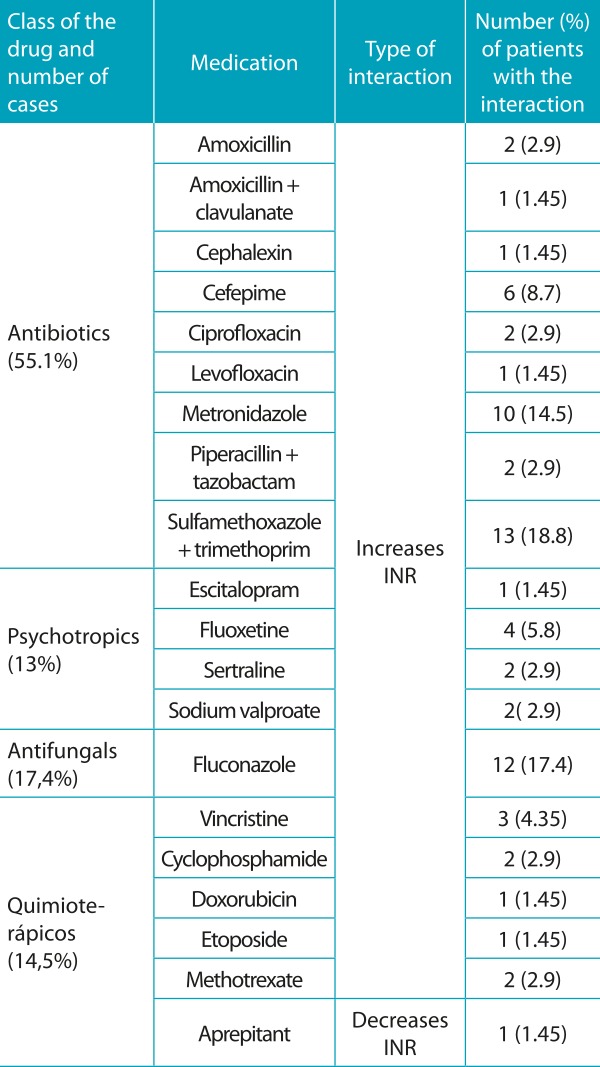
Potentially dangerous drug interactions found (69) in the period of anticoagulation, classified as severe, with good documentation.

INR: International normalized ratio.

## DISCUSSION

All patients presented at least one risk factor associated with VTE. The most common risk factors were CVC, cancer and use of TPN. The use of CVC was the most frequent factor, as demonstrated in the study by Van Ommen et al., with neonates (94%).^13^ The frequency of VTE related with catheter observed in the systematic review by Vidal et al., which included 3,128 patients aged <18 years, was 0.20 (95%CI 0.16-0.24).^3^ Andrew et al. verified that more than 50% of the VTE in children, which occurred in the upper venous network, were a result of the use of CVC,^12^ which calls our attention as to the location of VTEs in our study. More than half of the patients presented with thrombosis in the upper venous network. The mechanisms that can explain VTE related with CVC include lesions to the vessel wall caused by the CVC itself or by the infusion of substances like TPN and chemotherapy through the catheter, blockage of the blood flow or the material the catheter is made of.^3^ ^,^ ^16^

According to the literature, the risk of VTE in children with cancer is 1.52 per 1,000 children, whereas in children without the clinical condition the risk is reduced to 0.06/1.000.^17^ The factors that contribute with the increased risk of VTE in patients with cancer include changes in the hemostatic system as a consequence of the tumor, immobilization, need for frequent surgeries, chemotherapy (usually post-thrombosis), infections secondary to immunosuppression, as well as the frequent need of CVC by these patients.^6^

Besides the risks related with the use of CVC, the intravenous mixture of TPN may lead to a hypercoagulability state by the activation of pro-anticoagulant factors.^18^ As consequence, the proportion of VTE related with CVC in children with cancer (50%)^6^ is lower than in children using TPN (75%).^19^ The prophylactic use of warfarin in these patients has reduced the number of events.^20^

It is worth to mention that the effect of warfarin in the first days of treatment may not correspond to its plasmatic level, once this effect is related to the time required to exhaust the vitamin K-dependent clotting factors, and each factor has a different half life time.^21^ The consecutive increase in doses recommended in nomograms, such as for Andrew et al. ^22^ in the first days, according to the INR value, may increase the risk of INRs above the therapeutic target, and, therefore, the risk of bleeding. Besides, the potential interactions found increase the value of INR. Despite the known interaction of warfarin with antibiotics, psychotropic antifungals and chemotherapeutics, generally none of these is discontinued, and the monitoring of INR is the conduct recommended by the literature.^15^

Warfarin is available in the market exclusive in the form of pills, so it is necessary to adapt the doses by fractioning them and by deriving the pharmaceutical form. This procedure leads to variations in the process of preparation, especially when there is change from the manipulator, which can make anticoagulation difficult.

The concomitant use of UFH and/or LMWH, observed in some cases, is recommended by the American College of Chest Physicians, at least in the five first days or until reaching INR of 2.^8^

The doses of warfarin required for anticoagulation depend on age.^7^ ^,^ ^22^ The mechanisms according to which age influences the anticoagulation process are not clear.^11^ In this study, as in others that were published,^7^ ^,^ ^22^ there was an inverse relationship between age and dose of anticoagulation. Prospective studies reported that older children and adolescents remained with the INR on the target for a longer period of time (53-62%),^7^ ^,^ ^23^ however, this investigation demonstrated that younger children remained longer in the therapeutic range when compared to the other groups. One factor that can explain this discrepant result is the higher level of control of INR, with more frequent requests for PT tests, verified for patients younger than 1 year of age.

Throughout childhood, there are many variations in the amount of vitamin K present in the children’s diet. Maternal milk has small quantities, whereas the commercial children’s preparations contain higher levels.^24^ Patients with severe diseases, who need an enteral or parenteral diet, receive vitamin K supplement, whereas for older children with an oral diet, the levels of vitamin K depend on the type of food consumed. Two patients using TPN also received a weekly replacement of vitamin K while using warfarin. This fact can explain the longer time required for anticoagulation.^7^

Besides the need to use TPN, patients with SBS present reduced portions of the intestine, which may make it difficult to absorb nutrients and medication; therefore, this might be another factor that compromises the treatment negatively. Despite the report of resistance to the absorption of warfarin,^25^ ^,^ ^26^ patients with SBS did not present expressive differences in relation to the other groups as to the maintenance of therapy in the recommended interval, even if a larger dose was required to reach and maintain the target. A small study conducted with eight patients who had SBS using prolonged TPN indicated that the use of warfarin is safe, and that the TTR of 51.1% is not different from pediatric studies.^27^

Oncologic patients had the lowest INRs in the recommended therapeutic range (23.5%). The handling of the neoplasm therapy leads to many interruptions in the administration of warfarin, which usually makes anticoagulation difficult, since it takes longer for the INR to return to the therapeutic target.^28^

In three patients, the recurrence of thrombosis was verified (10.3%), and only one during the use of medication. The frequency found in this study was higher than that reported in the Netherlands (7.0%), and lower than the one described in Canada (18.5%).^12^ ^,^ ^13^ Even with the tendency found in all patients analyzed, to maintain the INRs below the recommended therapeutic range, the only recurrence of thrombosis while using the medication took place when the INR was supratherapeutic. In 2998, Masicotte et al. showed that children anticoagulated with vitamin K antagonist, such as warfarin, presented 25% less capacity than adults to produce thrombin, and observed that the concentration of endogenous thrombin markers and prothrombin fragments 1 and 2 was lower in children than in adults with the same INR value.^29^ These findings lead to the possibility that a lower therapeutic range than that recommended may be effective and safe in pediatrics, but further clinical studies in pediatric patients are required to establish the ideal therapeutic range of anticoagulation.

The limitations of this study have been related with the low number of cases and with the wide age group range of the patients. However, the intention was to include all patients assisted at the institution in the study period. The use of data obtained with the retrospective collection of medical records should also be considered, once the literature reports record problems associated with health care.

Despite these limitations, it was possible to verify that pediatric patients who presented with SBS and use TPN need significantly higher doses of warfarin than the other patients, in order to reach and maintain anticoagulation. The data in this study are in accordance with the literature, since it suggests that the doses of the drug and the time for anticoagulation vary according to the patient’s clinical condition, age group, route of drug administration and use of parenteral nutrition. The use of warfarin in pediatrics is complex because of the several factors that compromise anticoagulant therapy, reinforcing the need to elaborate protocols guiding clinical practice.
